# Viviendo entre dos culturas: Evaluating the psychometric properties of the Psychological Acculturation Scale among Latinx caregivers

**DOI:** 10.1371/journal.pone.0355228

**Published:** 2026-08-06

**Authors:** Alejandro L. Vázquez, Cynthia M. Navarro Flores, Lucybel Mendez, Jasmine Coleman, Gabriela A. Nagy, Araceli Garcia, Melanie M. Domenech Rodríguez

**Affiliations:** 1 Department of Psychology and Neuroscience, University of Tennessee, Knoxville, Knoxville, Tennessee, United States of America; 2 Department of Psychology and Brain Sciences, University of Wisconsin – Milwaukee, Milwaukee, Wisconsin, United States of America; 3 Department of Psychology, Utah State University, Logan, Utah, United States of America; PLOS: Public Library of Science, UNITED KINGDOM OF GREAT BRITAIN AND NORTHERN IRELAND

## Abstract

This study evaluated the structure, internal consistency, language-based measurement invariance, and construct validity of the Psychological Acculturation Scale (PAS), which assesses psychological acculturation via emotional attachment and belonging-based indicators. Analyses were conducted on a sample of 511 Latinx caregivers of youth with emotional/behavioral problems living in the United States (U.S.). Caregivers provided survey data either in English or Spanish on key sociodemographic factors (e.g., age, binary-gender, generational status in the U.S., language preference) and on psychological acculturation. Findings suggest that the PAS unidimensional factor structure had excellent internal consistency (α = .96, English α = .96, Spanish α = .90) and strong factor loadings (.75−.88) but fit indices for a confirmatory factor analysis were mixed. Post-hoc exploratory factor analyses were conducted to determine whether the model fit could be improved through the identification of a multidimensional structure, which uncovered two factors: (a) cultural identity and social belonging, and (b) cultural competence and familiarity. However, the two-factor solution fit better but had a high degree of overlap/redundancy (covariance = .923). A bifactor analysis suggest that the cultural identity and social belonging scale was unstable after accounting for the global scale and associated fit indices supported a unidimensional structure. The PAS unidimensional structure did not achieve configural invariance across English/Spanish versions, which suggest that differences in scores between language forms should be interpreted with caution. Our findings support the cautious use of the PAS as a brief unidimensional proxy measure of psychological acculturation to facilitate research with English/Spanish speaking Latinx caregivers.

## Introduction

Acculturation is associated with a wide variety of mental health outcomes and help-seeking behaviors among Latinx caregivers of youth (i.e., non-gender binary term denoting Latin American descent) [1–3]. Acculturation describes the process of navigating and integrating the distinct norms of one’s culture and the host culture [4]. As many Latinx youth and caregivers are immigrants and undergo the process of acculturation, understanding differences in acculturation level among the Latinx community and the measures used to assess this process is essential to unpack within-group differences in vulnerability, resilience to stressors, and cultural influences on mental health service utilization [1,2]. While theory highlights both behavioral (e.g., language, media use, music selection, holidays celebrated, types of food consumed) and psychological dimensions of acculturation (e.g., sense of emotional attachment and belonging), research on mental health outcomes and help-seeking associated with acculturation have largely emphasized behavioral indicators among Latinxs that may be less proximal to psychological outcomes relative to measures assessing phenomenological factors such as cultural emotional attachment and belong [5]. The Psychological Acculturation Scale (PAS) was developed to address this gap, but adoption has been limited due to a lack of formal psychometric evaluations of this scale, possibly limiting its inclusion in psychological research [6]. The present study sought to address this measurement gap by psychometrically evaluating the structure, construct validity, and measurement invariance between the English and Spanish PAS forms within a diverse sample of Latinx caregivers of youth.

### Acculturation, mental health, and help-seeking

The dimensionality of acculturation constructs has received significant research attention [7,8]. Historical conceptualizations of acculturation have often characterized acculturation as a unidimensional process, with individuals being on a continuum ranging from unacculturated to assimilated [6,9,10]. Within this perspective, higher acculturation to the host culture results in the loss of an individual’s sense of orientation and affiliation with their culture of origin [10]. Alternatively, scholars have conceptualized acculturation as a bidimensional phenomenon whereby there are parallel processes—the maintenance of one’s culture as well as the adoption of the host culture—resulting in four distinct acculturation categorizations, namely integration, assimilation, separation, and marginalization [11]. Bidimensional frameworks view cultural maintenance and acculturation to the host culture as conceptually independent [9]. Research has also conceptualized acculturation as a multidimensional process that occurs across various life domains (e.g., cultural behaviors, values, attachment, identity, belonging) [7]. Further, researchers have drawn attention to the need to consider limitations associated with conceptualizing the acculturation process in host-heritage dichotomies within pluralistic societies, as culture extends beyond traditional racial/ethnic orientations (e.g., sexual orientation, gender identity, nationality, disability), which has resulted in the development of integrative models that address various cultural orientations [12].

Approaches to conceptualizing acculturation may focus on cultural specificity or cultural fairness. The cultural specificity approach emphasizes group-specific experiences (e.g., identity, values, pride, identification) for a target population (e.g., Latinxs, Asians) [4,9,10]. A cultural fairness approach attempts to promote cross-group comparisons by using generalizable indicators of the acculturation process but often emphasizing behavioral indicators that may be more distal influences on mental health outcomes relative to psychological aspects of acculturation [4,9,10].

The process by which acculturation impacts mental health outcomes varies as a result of acculturative stress [13]. Acculturative stress refers to discomfort emerging from an individuals’ attempt to reconcile differences in language, norms, family expectations, and social roles between their origin and host country [13]. These challenges may result in individuals experiencing identity conflict, perceived cultural marginalization/discrimination, and/or pressure to assimilate, which can contribute to the development of mental health problems [1,3,14].

Acculturation may also shape help-seeking behaviors. For example, Latinx caregivers experiencing elevated acculturative stress may need mental health supports but may be less likely to seek support due to stigma, mistrust of institutions, limited culturally responsive care, and/or differing cultural beliefs about causes and treatment of mental health problems [1,3,14]. Alternatively, higher levels of bicultural integration may facilitate help-seeking by increasing familiarity with available mental health services, congruence between available treatments and perceived causes of mental health problems, and comfort navigating healthcare systems [15,16].

Research linking acculturation to mental health outcomes has largely emphasized behavioral measures, limiting our understanding of associations between psychological acculturation, acculturative stress, and related outcomes [7,17]. The underutilization of psychological acculturation measurements, like the PAS, may be due to the absence of formal psychometric evaluations of this measure, which may influence decisions regarding its inclusion in psychological research. Addressing this methodological limitation is crucial as measures of psychological acculturation may capture an internal cultural orientation process that is more proximally related to psychological functioning and outcomes relative to external behavioral indicators of acculturation [5].

### Measures of acculturation

Several unidimensional, bidimensional, and multidimensional measures of acculturation have been developed and/or psychometrically evaluated but largely emphasize behavioral indicators of acculturation and focus on specific target populations [4,10]. Unidimensional measures often assess acculturation as a continuum between heritage and host culture [4,10]. These measures frequently assess language use/preferences, cultural heritage or pride, media use, ethnic identity, classification, behaviors, interactions, values, and/or attitudes among other factors (e.g., Acculturation Rating Scale for Mexican Americans, Caetano Scale, Anderson Scale) [4,10]. Bidimensional measures often include separate subscales representing acculturation and enculturation formed by indicators representing language use, language proficiency, and electronic media use among other factors (e.g., Bidimensional Acculturation Scale; Acculturation Attitude Scale-Revised) [9,10]. Multidimensional measures commonly assess acculturation across various subscales including language use/preferences, ethnic identity, classification, cultural heritage, behaviors, interactions, values, attitudes, and/or interactions (e.g., Acculturation Rating Scale for Mexican Americans II, Hazuda Scale) [10]. More recently, multi-ethnic and multi-dimensional acculturation measures have been developed such as the Multi-Ethnic Acculturation Scale, which assesses Latinx and American cultural orientation separately with response being agreement with statements reflecting various behavioral and social indicators of acculturation (i.e., ranging from completely disagree [1] to completely agree [5]) [18]. While a wide variety of acculturation measures are available, few assess Latinx individuals’ senses of emotional attachment and belonging with their heritage and/or host cultures.

### Psychological acculturation scale

The PAS was developed to provide a complementary measure to behavioral-based scales, which focus on emotional-attachment and belonging-based acculturation [6]. The PAS assesses an individual’s psychological attachment and sense of belonging across a unidimensional continuum of emotional identification between Latinx/Hispanic and Anglo-American cultures and incorporates a bicultural middle group on this continuum influenced by Berry’s Model of Acculturation [6,11]. Although bidimensional measures are considered the standard in the field of psychology, meta-analytic research suggests that subscales comprising these measures have a weak negative correlation suggesting some loss of heritage culture through contact with the host culture, consistent with a unidimensional conceptualization [8]. It is also important to note that bidimensional measures often have a greater measurement burden relative to unidimensional measures that are composed of fewer items [4,10]. As unidimensional measures are associated with acculturation proxies (i.e., cultural preferences, knowledge, ethnic identification, generational status) in a similar manner to bidimensional measures, researchers have recommended their use as an inexpensive proxy acculturation measure and recommend bidimensional measures for deeper theoretical exploration [19]. Thus, the PAS can increase the feasibility of research due to its low measurement burden (i.e., 10 items) and should be viewed as a proxy measure for the acculturation process.

As a result of addressing a crucial aspect of psychological acculturation, the PAS has been used in diverse populations (e.g., immigrants from various countries, older adults, caregivers, Puerto Ricans) and referenced in numerous research articles [5]. Overall, psychological acculturation, as measured by the PAS, has been linked to various health behaviors and outcomes (e.g., criminal victimization, intent to breastfeed, prenatal anxiety, allostatic load, acculturation stress) [5,20–23]. For example, in a predominantly Puerto Rican sample of expectant mothers in the United States (U.S.) ages 16–30, Barcelona de Mendoza and colleagues found that higher levels of acculturation was related to lower odds of intent to combination feed (breastfeed and formula feed) [21]. Leveraging a similar sample, Barcelona de Mendoza and colleagues reported that PAS-derived bicultural acculturation was inversely associated with prenatal anxiety [22]. Further, Gassman-Pines and Skinner showed that higher levels of parental acculturation were inversely related to various parenting behaviors (e.g., neglect-indifference parenting) in a sample of Mexican immigrant families [24]. López-Cepero and colleagues also found that individuals with lower psychological acculturation were more likely to have a higher allostatic load (i.e., physiological adaptive response to cumulative stress) in a sample of adults aged 45–75, which is known to increase risk for physical and mental health problems [23]. Lastly, Capielo and colleagues found that Puerto Rican adults with low psychological acculturation living in Central Florida experienced more acculturation stress relative to those with higher acculturation to U.S. culture [5].

Despite its utility and use in the literature, a more comprehensive psychometric evaluation of the PAS has yet to be conducted beyond the Principal Component Analysis (PCA) and construct validation performed as part of the original measure development study [6]. This represents a significant gap in the psychometric literature that limits the impact of this tool in improving our understanding of acculturation processes. The present study sought to address this gap by confirming the structure, internal consistency, language-based measurement invariance, and construct validity of the PAS to aid mental health research with Latinx immigrant populations.

Information regarding the structure and validity of the PAS is largely based on the original development study conducted by Tropp and colleagues, which consisted of three studies published together [6]. The first study included 36 adults ages 13−58 living in the U.S. from various countries of origin (i.e., U.S., Puerto Rico, Mexico, and other Latin American countries). Respondents completed both the English and Spanish forms of the measure, which demonstrated strong mean correlations with each other (*r* = .94). Notably, although responses on individual items between the language forms were also strongly correlated (*r* = .70−.92), two specific items had lower correlations between forms (“In what culture(s) do you feel confident that you know how to act?” [*r* = .37]; “In what culture(s) do you know what is expected of a person in various situations?” [*r* = .64]). These lower correlations may signal some variability in factor loadings for items between language forms. This first study also found that both language versions had excellent internal consistency (English Cronbach’s α = .85; Spanish α = .83).

The second study performed a PCA of the measure utilizing a sample of Puerto Rican and U.S.-born adults (ages 12–58; *n* = 107). Results indicated that the PAS scale had a single factor, which explained 51% of the variance, with no additional identified factors (additional factors resulted in eigenvalues less than one). This study also reaffirmed that both language versions of the PAS had excellent internal consistency (English Cronbach’s α = .83; Spanish α = .90). Further, findings demonstrated that the PAS was related to proxies of acculturation in expected ways (i.e., higher acculturation among those born in Puerto Rico relative to those born on mainland; lower acculturation among those who chose to complete the survey in Spanish).

The third study focused on validating the PAS within a sample of caregivers (ages 27–57; *n* = 228) and their adolescents (ages 13–14; *n* = 247). In this study, PCAs conducted on the caregiver and adolescent samples also suggested that the PAS was represented by a single factor that explained a range of 55–56% of the variance. Findings further highlighted expected differences in acculturation levels. For instance, lower acculturation on the PAS was related to being born in Puerto Rico relative to the U.S. mainland and selection or preference for Spanish relative to English in the survey.

The foundational evaluation of the PAS conducted by Tropp and colleagues was followed up by Stevens and colleagues, who validated a Dutch language and culturally adapted version of the measure for Moroccan adolescents (*M*_age_ = 14.2; *n* = 783) and adults (*M*_age_ = 40; *n* = 387) living in the Netherlands [6,25]. Stevens and colleagues adapted the scale by selecting six items that assess emotional attachment and belonging, and duplicated these questions to measure psychological acculturation to Dutch and Moroccan culture separately, which added a bidimensional component to the scale [25]. Using this adapted version of the PAS, a Confirmatory Factor Analysis (CFA) established that a bidimensional model representing the aforementioned cultures fit the data better than a single unidimensional factor. Although this study further examined the structure and external validity of the PAS, research is still needed to determine the internal consistency, construct validity, and language-based measurement invariance of the PAS in larger and more diverse samples within the U.S.

### The present study

The present study sought to address the need for a comprehensive psychometric evaluation of the original English and Spanish PAS forms. Our aims were to a) confirm the structure of the PAS, b) evaluate internal consistency, c) explore language-based measurement invariance, and d) establish construct validity of the PAS within a diverse sample of Latinx caregivers. Based on prior research by Tropp and colleagues, we hypothesized that the PAS would be best represented by a single factor and would have good internal consistency [6]. Measures of language and generational status were expected to be related to higher acculturation scores in expected ways (i.e., greater preferences for English, selection of the English Survey, and greater generations in the U.S.) [6]. The unidimensional global factor representing the PAS was not expected to have metric invariance due to previously documented differences in item correlations between responses on the English and Spanish forms [6].

## Materials and methods

The present study utilized data from the second phase of the Pathways to Latinx Mental Health study, which aimed to identify factors that can promote mental health service parity for Latinx youths. The first phase examined youth mental health service utilization patterns and correlates among Latinx caregivers [3,26], while the second phase focused on Latinx caregivers’ perceptions related to help-seeking (e.g., telepsychology barriers, mental health etiologies, stigma, coping, psychological acculturation) [14,27]. Participants were recruited between August 24, 2021 and April 20, 2022 via a QuestionPro survey panel (data and R script are available through Open Science Framework; osf.io/d9n3b). Survey panels include individuals who are interested in being contacted about participating in research. QuestionPro emailed panel members a link explaining the study’s purpose, time commitment, and compensation. Eligible caregivers were those who identified as Latinx, lived in the U.S., and had at least one child ages 6–18 with emotional or behavioral problems. Caregivers who met the inclusion criteria, as determined by screening questions, were asked to read a letter of information and provided consent before continuing to the survey. Caregivers who were eligible and consented to participate completed a 30-minute online survey in English or Spanish. QuestionPro charged a $ 20 fee to the researchers for each participant who provided valid and complete responses and compensated participants in points that could be redeemed for rewards. Contrasting methods were used to ensure data integrity including attention checks, directed queries, open-ended responses, response time and pattern assessments, reCAPTCHA, and internet protocol (IP) address screening to prevent repeat submissions [28]. The protocol for the original study was approved by the Utah State University – Institutional Review Board (protocol #12087). Analysis of extant data for the present study was approved by the University of Tennessee, Knoxville – Institutional Review Board.

Of the 6,439 individuals who opened the survey (English = 3,007; Spanish = 3,432), 1,367 met eligibility criteria; 658 were excluded for poor-quality data and 193 were excluded for incomplete surveys. Five participants did not consent to participate and were not administered the survey. The final sample consisted of 511 Latinx caregivers of youths (English = 275, Spanish = 236).

### Measures

#### Sociodemographic.

Participants were asked to report their demographic information (i.e., age, binary-gender, generation status in the U.S., preferred language, relationship to youth, education). Two additional measures were used to capture racial and socioeconomic diversity to characterize the sample. Participants completed the MacArthur Scale of Subjective Social Status, which presents participants with a picture of a ladder with 10 rungs with responses ranging from the top rung 10 (*highest socioeconomic status in their community*) to 1 for the lowest rung (*the worse off in their community*) [29]. Participants were also shown a figure displaying 10 hands with shades of skin color ranging from 1 (*lightest*) to 10 (*darkest*) and were asked to select the skin tone that most closely matched their own [30].

The measures used to assess sociodemographic factors were translated from English to Spanish using a committee approach where one team member forward translated the measure from English to Spanish, another back translated the measure to English, and a third resolved discrepancies for the final measures [6,31]. Consistent with the original development study of the PAS, indicators representing proxies to acculturation, such as language selection (i.e., selected survey language), preferred language, and generation in the U.S., were used as indicators of construct validity [6].

#### Acculturation.

The PAS is a 10-item measure assessing an individual’s psychological attachment to differing cultural contexts [6]. Both English and Spanish language PAS forms used for the present study can be accessed through S1 File. Responses to these items were rated on a 9-point scale ranging from 1 (*Only Hispanic/Latino*) to 9 (*Only Anglo/American*). *Bicultural orientation* is represented by the midpoint value of each question (i.e., response of 5). A global mean score of PAS items can be calculated by averaging all items, with a higher score representing greater acculturation to Anglo/American culture. Participants were prompted:

*The following questions ask about how you feel about your Latino identity and heritage in relation to the majority “Anglo American” cultural group. Please provide your best answer to each item.*

### Analytic plan

#### A priori analysis.

The characteristics of the sample and PAS-related descriptive statistics were first examined using methods that were appropriate for variable type and distribution of responses (i.e., Analysis of Variance [ANOVA], Pearson’s bivariate correlations). The structure of the PAS was examined using CFA with the Maximum Likelihood estimator, as items were normally distributed. First, a global model was fitted with all scale items loading onto a single factor using responses from both language versions of the PAS. Model fit was assessed based on approximation to recommended cut-offs for fit indices: Root Mean Square Error of Approximation (RMSEA; good < .06, acceptable < .08, poor fit > .10), Comparative Fit Index (CFI; ≥ .95 good fit), Tucker-Lewis Index (TLI; good fit ≥ .95), and Standardized Root Mean Square Residual (SRMR; acceptable fit ≤ .08 [32]).

#### Post-hoc analysis.

As the unidimensional model had mixed findings related to fit, Horn’s parallel analysis with the Maximum Likelihood estimator was used to determine whether additional factors may be present in the scale that may improve model fit. An Exploratory Factor Analysis (EFA) using Pearson correlations and a Varimax rotation was used to obtain factor loadings and associated eigenvalues for the PAS. While Varimax, an orthogonal rotation, assumes uncorrelated factors, this approach was used as it provides easier-to-interpret simple structures (i.e., prevents cross-loadings). A post-hoc EFA scree test was then conducted to examine eigenvalues above one and identify the optimal factor solution. A subsequent CFA was fitted to compare the unidimensional to a multidimensional solution based on relative fit indices and a chi-square difference test. An orthogonal bifactor model was then estimated with scale items fitted onto the global factor and two-factor solutions simultaneously to determine whether a structure beyond a unidimensional factor was justified [33]. Several indices were used to evaluate the bifactor model including Explained Common Variance (ECV > .70–.80 strong general factor), Coefficient Omega (ω ≥ .70 suggest a reliable composite), and Coefficient Omega Hierarchical (ωH > .80 total score is essentially unidimensional) [34]. Language-based performance for the optimal PAS factor solution was then assessed in a stepwise manner to determine whether the English and Spanish forms were measurement invariant (i.e., configural, metric, scalar, strict; Jeong & Lee, 2019). Construct validity for the PAS scale was established by examining associations with indicators representing participant survey language selection, language preference, and generational status in the U.S. using Analysis of Variance (ANOVA) [35].

## Results

On average, participants were nearly 39 years old (*M* = 38.76, *SD* = 6.68), predominantly women (61.6%, *n* = 315), frequently first (38.0%, *n* = 194) or second (31.9%, *n* = 163) generation in the U.S., many had equal preference for English and Spanish (40.9%. *n* = 209), were largely biological parent to a youth (95.3%, *n* = 487), commonly had a post-high school education (67.3%, *n* = 344), and reported moderate to high subjective economic status (*M* = 6.41, *SD* = 1.86). Participants who completed the Spanish survey were more likely to be women, had darker skin tone, had greater representation of individuals who were first generation in the U.S., were more likely to prefer the Spanish language, and had lower education and subjective socioeconomic status relative to those who completed the English survey. Participants did not significantly differ between surveys in age. See Table 1 for sample characteristics by survey language. Participants generally reported lower acculturation on the PAS on the Spanish survey relative to the English survey. See Table 2 for PAS-related descriptive statistics. Pearson bivariate correlations suggested that all PAS items were generally positively correlated with each other, with effects ranging from moderate (*r* = .58) to high (*r* = .81; *p* < .001) across language forms. Items were approximately normally distributed, kurtosis and skewness were within acceptable limits (<−1 or +1), and the relations between items were linear based on scatterplots. See S1 Fig for the PAS item correlation panel.

**Table 1:** Sample characteristics by survey language.

Characteristics	Total (*n* = 511)	English (*n* = 275)	Spanish (*n* = 236)	*p*-value
**Age**	38.76 (6.68)	38.24 (6.83)	39.37 (6.46)	
**Binary-gender**				< .001
Men	196 (38.4%)	127 (46.2%)	69 (29.2%)	
Women	315 (61.6%)	148 (53.8%)	167 (70.8%)	
**Skin tone**	2.50 (1.32)	2.36 (1.34)	2.67 (1.27)	.001
**Generation in the U.S.**				< .001
1	194 (38%)	40 (14.5%)	154 (65.3%)	
2	163 (31.9%)	102 (37.1%)	61 (25.8%)	
3	65 (12.7%)	55 (20%)	10 (4.2%)	
4+	89 (17.4%)	78 (28.4%)	11 (4.7%)	
**Preferred language**				< .001
Only English	89 (17.4%)	76 (27.6%)	13 (5.5%)	
Mostly English	87 (17%)	66 (24%)	21 (8.9%)	
Equally English/Spanish	209 (40.9%)	113 (41.1%)	96 (40.7%)	
Mostly Spanish	99 (19.4%)	20 (7.3%)	79 (33.5%)	
Only Spanish	27 (5.3%)	0 (0%)	27 (11.4%)	
**Education**				< .001
Highschool equivalent or less	167 (32.7%)	65 (23.6%)	102 (43.2%)	
Post high school	344 (67.3%)	210 (76.4%)	134 (56.8%)	
**Subjective socioeconomic status**	6.41 (1.86)	6.64 (1.94)	6.13 (1.71)	< .001

Analysis of Variance (ANOVA) and chi-square test of independence were used for comparisons. Mean/frequency (standard deviation / %).

**Table 2:** Summary statistics for psychological acculturation scale items and scales.

Scales (Cronbach’s α) and items	Total (*N* = 511) *M (SD)*	English (*n* = 275) *M (SD)*	Spanish (*n* = 236) *M (SD)*	*p*-value
**Cultural identity and social belonging (Total α = .89, English α = .90, Spanish α = .87)**	**4.42 (1.86)**	**5.06 (1.77)**	**3.68 (1.68)**	**< .001**
1) With which group of people do you feel you share most of your beliefs and values?	4.43 (2.09)	5.11 (1.96)	3.64 (1.95)	< .001
2) With which group of people do you feel you have the most in common?	4.40 (2.07)	5.03 (1.96)	3.66 (1.95)	< .001
3) With which group of people do you feel most comfortable?	4.44 (2.00)	5.04 (1.89)	3.74 (1.89)	< .001
**Cultural competence and familiarity (Total α = .94, English α = .95, Spanish α = .84)**	**4.45 (1.79)**	**5.12 (1.71)**	**3.67 (1.54)**	**< .001**
4) In your opinion, which group of people best understands your ideas (your way of thinking)?	4.45 (2.08)	5.07 (1.93)	3.73 (2.03)	< .001
5) Which culture do you feel proud to be a part of?	4.27 (2.08)	4.97 (1.95)	3.45 (1.92)	< .001
6) In what culture do you know how things are done and feel that you can do them easily?	4.41 (2.11)	5.13 (1.97)	3.56 (1.95)	< .001
7) In what culture do you feel confident you know how to act?	4.57 (2.04)	5.17 (1.97)	3.87 (1.90)	< .001
8) In your opinion, which group of people do you understand best?	4.49 (2.09)	5.16 (1.94)	3.70 (1.97)	< .001
9) In what culture do you know what is expected of a person in various situations?	4.64 (1.98)	5.28 (1.81)	3.90 (1.91)	< .001
10) Which culture do you know the most about (for example: its history, traditions, and customs)?	4.34 (2.19)	5.09 (2.07)	3.46 (2.00)	< .001
**Global factor (α = .96, English α = .96, Spanish α = .90)**	**4.44 (1.75)**	**5.10 (1.68)**	**3.67 (1.50)**	**< .001**

*p*-values represent mean differences for each item by language using Analysis of Variance (ANOVA).

### Measurement structure and internal consistency

**Fig 1:**
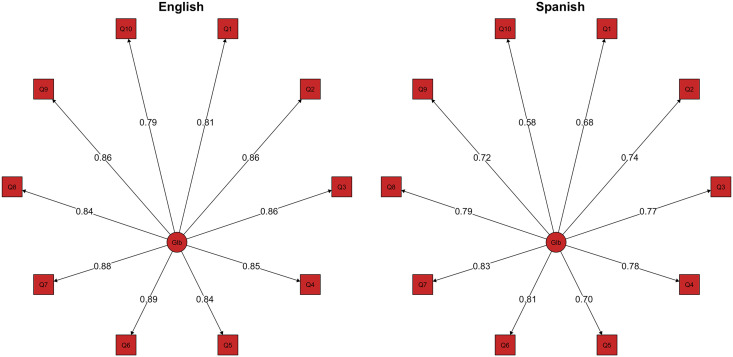
Global factor model loading by language (*N* = 511). English (*n* = 275), Spanish (*n* = 236).

First, a global CFA model was fitted with all scale items loading onto a global factor using responses from both language versions of the PAS. The model fit for the global factor solution was mixed with two metrics suggesting marginally poor fit (i.e., RMSEA, TLI), and two suggest a good fit (i.e., CFI, SRMR). Despite the mixed fit indices, the global factor model had excellent internal consistency (α = .96) and strong item loadings (ranging from .75−.88). See Table 3 for overall sample factor loadings and Fig 1 for the global factor model loadings by language version.

**Table 3:** Fit statistics for global factor, two-factor, bifactor, and language invariance models (N = 511).

Model	*χ* ^*2*^	*df*	*p*-value	RMSEA [90% CI]	SRMR	CFI	TLI
**Measurement**							
Global factor	238.94	35	< .001	.107 [.094 – .120]	.030	.955	.942
Two-factor	153.36	34	< .001	.083 [.070 – .096]	.025	.974	.965
Bifactor ^a^	102.57	28	< .001	.072 [.058 – .087]	.018	.984	.974
**Invariance**							
Configural	313.71	70	< .001	.117 [.104 – .130]	.037	.941	.925

*χ*^*2*^ = chi-square statistic, *df* = degrees of freedom, RMSEA = Root Mean Square Error of Approximation, CFI = Comparative Fit Index, TLI = Tucker-Lewis Index, SRMR = Standardized Root Mean Square Residual. ^a^ Items loaded onto global and cultural competence and familiarity factors simultaneously.

Horn’s parallel analysis with the Maximum Likelihood estimator was then conducted to determine whether additional factors may be present in the scale that may improve model fit. The parallel analysis suggested that in our sample, the PAS may be represented by up to two factors (See S2 Fig.). An EFA was conducted to obtain factor loadings and associated eigenvalues for the PAS. The EFA identified two likely factors in the data representing (a) cultural identity and social belonging (items 1–3; eigenvalue = 3.98) and (b) cultural competence and familiarity (items 4–10; eigenvalue = 3.18). A post-hoc EFA scree test was then conducted, which suggested that a third factor may be warranted based on eigenvalues above one (See S3 Fig.). A three-factor EFA was explored but was rejected as the solution produced two factors represented by two items each. The two scales represented by two items introduce issues related to the reliability and stability of the constructs being measured and challenges related to CFA models that typically require at least three items per factor to provide sufficient degrees of freedom to estimate model parameters and latent variables.

A CFA model with the Maximum Likelihood estimator was then used to assess the two-factor solution identified by the EFA. The two-factor model fit was in the recommended range for all approximate fit indices (i.e., RMSEA, SRMR, CFI, TLI). A chi-squared difference test also suggested that the two-factor model fit the data significantly better than the global factor solution, *χ*^2^_difference_, (1) = 85.58, *p* < .001. In addition, the two-factor model had excellent internal consistency for cultural identity and social belonging (α = .94) and cultural competence and familiarity scales (α = .89) and had item loadings ranging from .75−.88. However, the covariance for these factors was high (.923), which suggest that they had high overlap and were potentially redundant. See Table 4 for overall global factor loadings and see S4 Fig for loadings by language forms.

**Table 4:** Overall sample factor loadings for Psychological Acculturation Scale (N = 511).

	Confirmatory factor analysis			Exploratory factor analysis	
Items	Global factor	Cultural identity and social belonging	Cultural competence and familiarity	Cultural identity and social belonging	Cultural competence and familiarity
1) With which group of people do you feel you share most of your beliefs and values?	.786	.829	–	.405	**.748**
2) With which group of people do you feel you have the most in common?	.830	.880	–	.457	**.758**
3) With which group of people do you feel most comfortable?	.839	.866	–	.537	**.665**
4) In your opinion, which group of people best understands your ideas (your way of thinking)?	.834	–	.826	**.593**	.588
5) Which culture do you feel proud to be a part of?	.811	–	.816	**.668**	.465
6) In what culture do you know how things are done and feel that you can do them easily?	.876	–	.884	**.756**	.466
7) In what culture do you feel confident you know how to act?	.872	–	.884	**.815**	.399
8) In your opinion, which group of people do you understand best?	.843	–	.849	**.707**	.469
9) In what culture do you know what is expected of a person in various situations?	.820	–	.827	**.608**	.460
10) Which culture do you know the most about (for example: its history, traditions, and customs)?	.745	–	.747	**.566**	.483

Bold are best factor loading for each factor in Exploratory Factor Analysis.

A bifactor model was then used to simultaneously load items onto the global and two-factor solutions resulted in negative variance. Item 3 within the cultural identity and social belonging scale was identified as have a large negative loading and variance. Several techniques were used to address this issue, including setting a new starting point and forcing the model to converge on an estimate above zero for item 3. These efforts were unsuccessful in correcting the negative variance, which suggest that the cultural identity and social belonging scale was unstable after accounting for the global factor. The bifactor model converged with a structure composed of the global scale and cultural competence and familiarity scale with fit indices generally in the recommended range (i.e., RMSEA, SRMR, CFI, TLI). A chi-squared difference test also suggested that the bifactor model fit the data significantly better than the two-factor solution, *χ*^2^_difference_, (6) = 50.79, *p* < .001. Factor loadings for the bifactor model ranged from .13 – .87 (See S5 Fig). The bifactor fit index suggest that 89.7% of the common variance between items was explained by the global factor (ECV = .897), 96.0% of the total score reflected common variance instead of measurement error (ω coefficient), and 89.4% of variance in total scores was attributed to the global factor (ωH). These findings suggest that the PAS was best represented by a unidimensional global factor as additional factors only accounted for limited unique variance.

### Measurement invariance

Language-based performance for the PAS global factor model was assessed in a stepwise manner to determine whether the English/Spanish forms were measurement invariant (i.e., configural, metric, scalar, strict; Jeong & Lee, 2019). First, a configural model with the global factor structure was fitted in both the English and Spanish forms, which had poor fit across indices relative to the freely estimated unidimensional model (RMSEA, SRMR, CFI, TLI). This finding suggest that the latent structure of the measure was different across language version of the PAS. As configural invariances was not achieved, additional invariance test were not conducted.

### Construct validity

**Fig 2:**
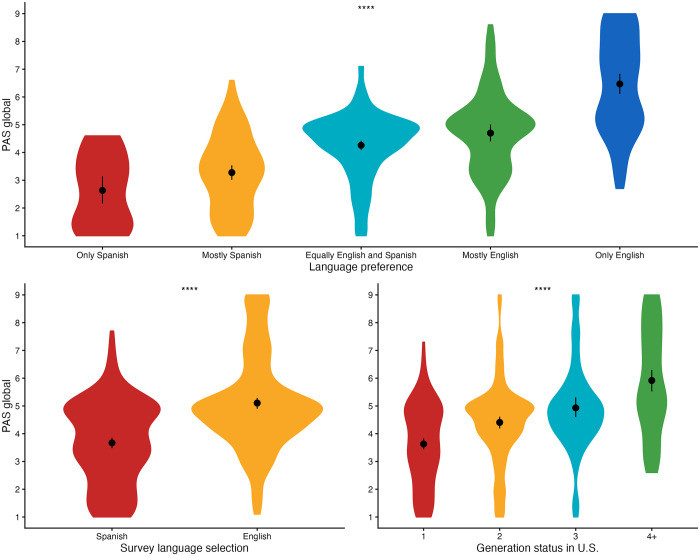
Psychological acculturation global scale associations with construct validity indicators. Analysis of Variance (ANOVA) were used for comparisons. Mean and standard deviation are displayed. p > .05 (no stars), p ≤ .05 (*), p ≤ .01 (**), p ≤ .001 (***), p ≤ .0001 (****). PAS = Psychological Acculturation Scale.

Differences in PAS scores were examined by participant language selection, language preference, and generational status in the U.S. using ANOVA. Global scores on the PAS increased (i.e., greater Anglo/American acculturation) in concordance with more generations within the U.S., greater preference towards English, and among those who selected the English survey. See Fig 2 for construct validity measures by the PAS global scale.

## Discussion

Psychological acculturation is a key process for Latinx immigrants that influences their vulnerability, resiliency, and mental health service utilization patterns [1,2,16]. The present study built on prior research that documented the development of the PAS by conducting a psychometric evaluation that further elucidated the scales’ structure, language-based measurement invariance, internal consistency, and construct validity [6]. While the original unidimensional solution for the PAS had good internal consistency, CFA analysis suggests that it had mixed findings regarding model fit, with some metrics suggesting marginal poor fit and others suggesting acceptable fit. A post-hoc EFA was conducted to attempt to improve model fit by undercovering a potential multidimensional structure, which resulted in the identification of two-factors representing a) cultural identity and social belonging, and b) cultural competence and familiarity. These factors were interpreted post-hoc based on the emotional and belonging-based indicators that were represented within each subscale and correspond with aspects of the acculturation process represented in the broader literature [4,6,9,10]. Evidence from a subsequent CFA showed that the two-factor structure had a better fit than the global factor solution while maintaining excellent internal consistency. However, a bifactor model uncovered instability in the cultural identity and social belonging scale after accounting for the global factor, and fit indices suggest that the PAS was best represented by global factor as the multidimensional structure did not explain a significant portion of the variance in PAS scores. These findings confirm the unidimensional factor structure proposed by Tropp and colleagues within a sample with greater Latinx diversity relative to the original PAS development study, which focused largely on Puerto Ricans [6].

In addition, expanding upon prior evidence that showed strong correlations between the English and Spanish versions of the scale [6], results from measurement invariance tests between these forms indicated that the PAS did not achieve configural invariance. In other words, the PAS structure functioned differently across English and Spanish forms in terms of factor structure. As a result, differences in scores between English and Spanish forms of the PAS should be interpreted with caution as this may represent differences in the latent construct and measurement error. This degree of measurement error for the PAS may be related to slight differences in interpretations of items or context, such as varying levels of exposure to the host or culture of origin. Further research is needed to determine potential sources of variability in measurement error between language forms. Thus, while the PAS represents a reliable proxy measure of acculturation, researchers examining PAS scores across respondents using English and Spanish forms should interpret differences with caution as it is expected to capture differences in psychological acculturation with some differential functioning of the measures across languages.

Lastly, the present study found expected associations between scores on the PAS and related proxies of acculturation, including behavioral and generational indicators. Specifically, the results indicated that higher acculturation scores were present for Latinx individuals who reported higher generation status, a preference for English as their primary language, and selected the English survey. These findings provide evidence for the construct validity of the PAS and are in alignment with evidence from the original development study, which found similar associations with proxies of acculturation [6].

### Implications

The findings presented herein add to the existing body of acculturation-related literature indicating that the PAS is a reliable measure. Our research highlights a need for caution when using the different language forms of this tool with Latinx adults. Moreover, the PAS centers on emotional and belonging-based indicators of acculturation, in contrast to many existing measures that predominantly center on behaviors, representing critical yet often overlooked dimensions of this construct. To this end, the novel focuses on emotions and identity, allowing for a more nuanced and comprehensive understanding of the acculturative experience, capturing the internal psychological processes that shape an individual’s sense of belonging, self-concept, and well-being within and across cultural contexts. As such, this tool could be utilized in conjunction with other acculturation tools that focus on behavioral indicators, thereby providing potentially more comprehensive and nuanced data on the acculturation processes among Latinx individuals, which is known to be heterogeneous and multifaceted. However, while the assessment of psychological acculturation may provide important information on internal cultural orientations that may be more proximal to psychological functioning, research is needed to determine the incremental contributions of using the PAS alongside more distal behavioral indicators in explaining within-group differences in mental health and help-seeking outcomes among Latinxs [5].

While bidimensional measures are considered the standard in the field of psychology, research suggests that acculturation and enculturation measures have a weak negative correlation suggesting some loss of heritage culture through contact with the host culture, consistent with a unidimensional conceptualization used in the development of the PAS [8]. The PAS may also increase the feasibility of acculturative research as it is composed of fewer items than most bidimensional measures [4,10]. Thus, while the unidimensional design of the PAS limits its use for research focusing on in-depth theoretical explorations of acculturative processes, this scale may facilitate research as a brief proxy measure of acculturation that can be used to examine within-group differences in mental health and help-seeking outcomes among Latinxs [19]. Future research should also adapt and evaluate a bidimensional version of the PAS that measures emotional attachment and belonging-based indicators of acculturation and enculturation separately to facilitate deeper theoretical research [25] These measures can help elucidate within-group differences in help-seeking behaviors and perceptual barriers to accessing care for youth among Latinx caregivers [3,14,26,27]. Additionally, future research is needed to further assess the validity of this tool, for example, its concurrent and discriminant validity. Lastly, given distinct acculturation trajectories within the heterogeneous Latinx community, future research could utilize latent profile analyses to identify profiles of psychological acculturation.

### Strengths and limitations

There are several important strengths of the study that are worth mentioning. First, the present study extended the scientific literature pertaining to the measurement of the acculturation of Latinx people using the PAS tool through the inclusion of structural analyses, construct validity, and language-based measurement invariance. Additionally, this study engaged a large and diverse sample of caregivers and deployed the survey in English and Spanish, which holds important implications for the generalizability of findings.

This study also has several limitations. First, the relatively small sample size for each language version of the PAS constrained our ability to separately validate factors identified by the EFA and subsequently evaluated these factors through a CFA and a bifactor model. In order to address this limitation, future research should confirm the present findings in a larger sample. Additionally, recruiting participants through QuestionPro required that we only use responses from participants who provided complete responses and high-quality data, which may have potentially introduced bias by excluding those hesitant to report on their child’s mental health and those who have difficulties with literacy [27]. Leveraging other recruitment methods such as in-person recruitment through Latinx serving community organizations would help address this limitation in future studies. The present study did not assess concurrent validity between the PAS and other measures of acculturation or associations with contextual factors that may influence participants’ reporting on the measure. Contemporary multi-ethnic acculturation frameworks describe non-linear and domain-specific acculturation processes, and potential mismatches between behavioral and psychological acculturation domains. For example, different domains of acculturation (e.g., cognitive, behavioral, identity-based) may change at different rates and in different directions over time [18]. As a result, it is recommended that future research should examine associations between the PAS and established measures of acculturation and contextual factors known to impact acculturation processes (e.g., geographic region, political climate) [8]. Also, this sample only focused on Latinx caregivers living in the U.S. who had a child with emotional or behavioral problems, limiting the external validity of the present findings. Future studies should investigate whether these results generalize to Latinx caregivers living outside of the U.S. and caregivers whose children do not have emotional or behavioral difficulties. Lastly, acculturation is a dynamic and context-sensitive process, and as a result responses to emotionally and identity-based items included in the PAS may be influenced by contextual or situational factors (e.g., caregiving stress) that reflect the caregiver's current psychological state. Future research should examine potential construct contamination resulting from contextual or situational factors that may influence reporting on the PAS such as caregiver strain.

### Conclusions

In sum, the present study advances the field of acculturation research by offering a psychometric evaluation of the PAS. Most measures of acculturation focus on assessing behavior-based indicators of this process [4,6,10]. Instead, the PAS focuses on capturing emotional and belonging-based indicators that serve as a proxy of the acculturation process and has been utilized in numerous research studies as a result [23,24]. However, despite its focus on distinct aspects of psychological acculturation and incremental value, a lack of psychometric evaluation of the PAS limits the measures’ potential inclusion and effectiveness in research. By psychometrically evaluating a measure assessing these understudied aspects of acculturation, the present study offers not only an important tool that may advance our understanding of the phenomenon of psychological acculturation but may also lead to insights that can help clinicians support Latinx caregivers undergoing the process of immigration and subsequent acculturation in the U.S.

## Supporting information

S1 Fig Bivariate Pearson correlations between PAS items (*N* = 511). * *p* < .05, **p < .010, ***p < .001.
(TIFF)

S2 Fig Parallel analysis for PAS items (*N* = 511). (TIFF)

S3 Fig Exploratory factor analysis scree plot (*N* = 511). (TIFF)

S4 Fig Two-factor model loadings by language (*N* = 511). English (*n* = 275), Spanish (*n* = 236).
(TIFF)

S5 Fig Bifactor model simultaneously loading items onto the global factor and cultural competence and familiarity subscale (*N* = 511). (TIFF)

S1 File Psychological acculturation scale English and Spanish forms. (DOCX)

## References

[1] Galvan T, Gudiño OG. Understanding Latinx youth mental health disparities by problem type: the role of caregiver culture. Psychol Serv. 2021;18(1):116–23. doi: 10.1037/ser0000365 31192675

[2] Kapke TL, Gerdes AC. Latino family participation in youth mental health services: treatment retention, engagement, and response. Clin Child Fam Psychol Rev. 2016 Dec 1;19(4):329–51. doi: 10.1007/s10567-016-0213-2 27585812

[3] Vázquez AL, Alvarez M de la C, Navarro Flores CM, González Vera JM, Barrett TS, Domenech Rodríguez MM. Youth mental health service preferences and utilization patterns among Latinx caregivers. Child Youth Services Rev. 2021;131:106258. doi: 10.1016/j.childyouth.2021.106258

[4] Wallace PM, Pomery EA, Latimer AE, Martinez JL, Salovey P. A review of acculturation measures and their utility in studies promoting latino health. Hisp J Behav Sci. 2010;32(1):37–54. doi: 10.1177/0739986309352341 20582238 PMC2891555

[5] Capielo C, Delgado-Romero EA, Stewart AE. A focus on an emerging Latina/o population: the role of psychological acculturation, acculturative stress, and coping on depression symptoms among central Florida Puerto Ricans. J Lat Psychol. 2015 Nov;3(4):209–23. doi: 10.1037/lat0000039

[6] Tropp LR, Erkut S, Coll CG, Alarcón O, García HAV. Psychological acculturation: development of a new measure for puerto ricans on the U.S. mainland. Educ Psychol Meas. 1999;59(2):351–67. doi: 10.1177/00131649921969794 21415932 PMC3057082

[7] Yoon E, Langrehr K, Ong LZ. Content analysis of acculturation research in counseling and counseling psychology: a 22-year review. J Couns Psychol. 2011;58(1):83–96. doi: 10.1037/a0021128 21142352

[8] Yoon E, Cabirou L, Galvin S, Hill L, Daskalova P, Bhang C, et al. A meta-analysis of acculturation and enculturation: bilinear, multidimensional, and context-dependent processes. Couns Psychol. 2020 Apr 15;48(3):342–76. doi: 10.1177/0011000019898583

[9] Celenk O, Van de Vijver FJR. Assessment of acculturation: issues and overview of measures. Online Readings in Psychology and Culture. 2011;8(1). doi: 10.9707/2307-0919.1105

[10] Thomson MD, Hoffman-Goetz L. Defining and measuring acculturation: a systematic review of public health studies with Hispanic populations in the United States. Soc Sci Med. 2009;69(7):983–91. doi: 10.1016/j.socscimed.2009.05.011 19525050

[11] Berry JW. Acculturation: a conceptual overview. in: acculturation and parent-child relationships. Routledge; 2006. 13–32. doi: 10.4324/9780415963589-2

[12] Garcia JG, DiNardo J, Nuñez MIL, Emmanuel D, Chan CD. The integrated acculturation model: expanding acculturation to cultural identities in addition to race and ethnicity. J Multicult Couns Devel. 2020;48(4):271–87. doi: 10.1002/jmcd.12199

[13] Bekteshi V, Kang S-W. Contextualizing acculturative stress among Latino immigrants in the United States: a systematic review. Ethn Health. 2020;25(6):897–914. doi: 10.1080/13557858.2018.1469733 29792072

[14] Vázquez AL, Domenech Rodríguez MM, Navarro Flores CM, Abreu RL, Barrett TS. La vergüenza [the shame]: Measuring affiliate stigma associated with youth mental health problems among Latinx caregivers. J Latinx Psychology. 2024;12(1):18–33. doi: 10.1037/lat0000235

[15] Srebnik D, Cauce AM, Baydar N. Help-seeking pathways for children and adolescents. J Emot Behav Disord. 1996;4(4):210–20. doi: 10.1177/106342669600400402

[16] Vázquez AL, Chou T, Helseth SA, Gudiño OG, Domenech Rodríguez MM. Juntos hacemos la diferencia [together we make the difference]: a network analysis of Latinx caregivers’ use of youth support services. Fam Process. 2024;63(2):788–802. doi: 10.1111/famp.12901 37277975 PMC10696132

[17] Yoon E, Chang C-T, Kim S, Clawson A, Cleary SE, Hansen M, et al. A meta-analysis of acculturation/enculturation and mental health. J Couns Psychol. 2013;60(1):15–30. doi: 10.1037/a0030652 23163612

[18] Goytizolo A. Psychometric properties of a new acculturation scale for Latinos/Hispanics. [Internet]. Florida Atlantic University; 2025. https://digitalcommons.fau.edu/etd_general/116

[19] Flannery WmP, Reise SP, Yu J. An empirical comparison of acculturation models. Pers Soc Psychol Bull. 2001;27(8):1035–45. doi: 10.1177/0146167201278010

[20] Adams WE, Todorova ILG, Falcón LM. Puerto Rican Victimization and Crime on the Mainland. Hisp J Behav Sci. 2015;37(1):59–74. doi: 10.1177/0739986314564149 33223605 PMC7679081

[21] Barcelona deMendozaV, Harville E, Theall K, Buekens P, Chasan‐Taber L. Acculturation and intention to breastfeed among a population of predominantly puerto rican women. Birth. 2016;43(1):78–85. doi: 10.1111/birt.12199 26554873 PMC4755899

[22] Barcelona de Mendoza V, Harville E, Theall K, Buekens P, Chasan-Taber L. Effects of acculturation on prenatal anxiety among Latina women. Arch Womens Ment Health. 2016;19(4):635–44. doi: 10.1007/s00737-016-0605-9 26790686 PMC4956601

[23] López-Cepero A, McClain AC, Rosal MC, Tucker KL, Mattei J. Examination of the allostatic load construct and its longitudinal association with health outcomes in the Boston Puerto Rican Health Study. Psychosom Med. 2022;84(1):104–15. doi: 10.1097/PSY.0000000000001013 34581702 PMC8678200

[24] Gassman-Pines A, Skinner AT. Psychological acculturation and parenting behaviors in mexican-immigrant families. J Fam Issues. 2018 Apr 11;39(5):1139–64. doi: 10.1177/0192513X16687001 29545656 PMC5846495

[25] Stevens GWJM, Pels TVM, Vollebergh WAM, Crijnen AAM. Patterns of psychological acculturation in adult and adolescent moroccan immigrants living in the Netherlands. J Cross-Cultural Psychol. 2004;35(6):689–704. doi: 10.1177/0022022104270111

[26] Vázquez AL, Navarro Flores CM, Alvarez M de la C, Domenech Rodríguez MM. Latinx caregivers’ perceived need for and utilization of youth telepsychology services during the coronavirus pandemic. J Latinx Psychol. 2021;9(4):284–98. doi: 10.1037/lat0000192

[27] Vázquez AL, Domenech Rodríguez MM, Cadenas GA, Barrett TS, Navarro Flores CM. Barriers to accessing telepsychology services questionnaire: structure and language-based performance in a sample of Latinx caregivers. Psychol Serv. 2024;21(1):50–64. doi: 10.1037/ser0000804 37856391

[28] Abbey JD, Meloy MG. Attention by design: using attention checks to detect inattentive respondents and improve data quality. J of Ops Management. 2017;53–56(1):63–70. doi: 10.1016/j.jom.2017.06.001

[29] Adler N, Stewart J. The MacArthur scale of subjective social status. 2007. https://sparqtools.org/wp-content/uploads/2022/10/MacArthur-Scale-of-Subjective-Social-Status-Adult-Version.pdf

[30] Rastogi M, Massey-Hastings N, Wieling E. Barriers to seeking mental health services in the latino/a community: a qualitative analysis. J Systemic Therap. 2012;31(4):1–17. doi: 10.1521/jsyt.2012.31.4.1

[31] Beaton DE, Bombardier C, Guillemin F, Ferraz MB. Guidelines for the process of cross-cultural adaptation of self-report measures. Spine (Phila Pa 1976). 2000;25(24):3186–91. doi: 10.1097/00007632-200012150-00014 11124735

[32] Hu L, Bentler PM. Cutoff criteria for fit indexes in covariance structure analysis: Conventional criteria versus new alternatives. Struct Equ Modeling. 1999 Jan;6(1):1–55. doi: 10.1080/10705519909540118

[33] Rodriguez A, Reise SP, Haviland MG. Applying bifactor statistical indices in the evaluation of psychological measures. J Pers Assess. 2016 May 3;98(3):223–37. doi: 10.1080/00223891.2015.1089249 26514921

[34] Reise SP, Moore TM, Haviland MG. Bifactor models and rotations: exploring the extent to which multidimensional data yield univocal scale scores. J Pers Assess. 2010;92(6):544–59. doi: 10.1080/00223891.2010.496477 20954056 PMC2981404

[35] Roth KB, Musci RJ, Eaton WW. Refining acculturation measures for health research: Latina/o heterogeneity in the National Latino and Asian American Study. Int J Methods Psychiatr Res. 2020;29(4):1–12. doi: 10.1002/mpr.1844 32845559 PMC7723201

