# Supplementary material for: Viviendo entre dos culturas: Evaluating the psychometric properties of the Psychological Acculturation Scale among Latinx caregivers
Source: PLoS One. 2026 Aug 6;21(8):e0355228. doi: 10.1371/journal.pone.0355228 (PMC13446657; doi:10.1371/journal.pone.0355228)
Supplement: Supplementary file 1 [file pone.0355228.s006.docx]

**Psychological Acculturation Scale**

**English Form**

The following questions ask about how you feel about your Latino identity and heritage in relation to the majority “Anglo American” cultural group. Please provide your best answer to each item.

| 1  Only Hispanics/Latinos | 2 | 3 | 4 | 5  Equally Hispanic/Latinos and Anglos (Americans) | 6 | 7 | 8 | 9  Only Anglos (Americans) |
| --- | --- | --- | --- | --- | --- | --- | --- | --- |

1) With which group of people do you feel you share most of your beliefs and values?

2) With which group of people do you feel you have the most in common?

3) With which group of people do you feel most comfortable?

4) In your opinion, which group of people best understands your ideas (your way of thinking)?

5) Which culture do you feel proud to be a part of?

6) In what culture do you know how things are done and feel that you can do them easily?

7) In what culture do you feel confident you know how to act?

8) In your opinion, which group of people do you understand best?

9) In what culture do you know what is expected of a person in various situations?

10) Which culture do you know the most about (for example: its history, traditions, and customs)?

**Psychological Acculturation Scale**

**Spanish Form**

Las siguientes preguntas son acerca de cómo se siente acerca de su identidad y herencia Latina en relación con el grupo cultural mayoritario "angloamericano." Por favor, proporcione sus mejores respuestas a cada pregunta.

| 1  Sólo Hispanos/Latinos | 2 | 3 | 4 | 5  Hispanos/Latinos y Anglos (Americanos) ambos por igual | 6 | 7 | 8 | 9  Sólo Anglos (Americanos) |
| --- | --- | --- | --- | --- | --- | --- | --- | --- |

1) ¿Con qué grupo de personas siente que comparte la mayoría de sus creencias y valores?

2) ¿Con qué grupo de personas siente que tiene lo más en común?

3) ¿Con qué grupo de personas se siente más cómodo(a)?

4) En su opinión, ¿qué grupo de personas entiende mejor sus ideas (su forma de pensar)?

5) ¿De qué cultura se siente orgulloso(a) de ser miembro?

6) ¿En qué cultura usted sabe cómo se hacen las cosas y siente que puede hacerlas con facilidad?

7) ¿En qué cultura se siente seguro(a) de que sabe cómo comportarse?

8) En su opinión, ¿a qué grupo de personas entiende mejor?

9) ¿En qué cultura sabe lo que se espera de una persona en varias situaciones?

10) ¿De qué cultura conoce más (por ejemplo: su historia, sus tradiciones, y sus costumbres)?
